# The interplay between H3K36 methylation and DNA methylation in cancer

**DOI:** 10.20892/j.issn.2095-3941.2023.0234

**Published:** 2023-08-18

**Authors:** Jiameng Dan, Zeling Du, Jinghong Zhang, Taiping Chen

**Affiliations:** 1 State Key Laboratory of Primate Biomedical Research, Institute of Primate Translational Medicine, Kunming University of Science and Technology, Kunming 650500, China; 2 Yunnan Key Laboratory of Primate Biomedical Research, Kunming 650500, China; 3 Department of Epigenetics and Molecular Carcinogenesis, The University of Texas MD Anderson Cancer Center, Houston 77030, USA; 4 Programs in Genetics and Epigenetics, The University of Texas MD Anderson Cancer Center UT Health Graduate School of Biomedical Sciences, Houston 77030, USA

Cancer refers to a diverse collection of diseases characterized by several well-established hallmarks, including the abilities to sustain proliferative signaling, evade growth suppressors, activate invasion and metastasis, enable replicative immortality, induce angiogenesis, and resist cell death^1^. Historically, genetic alterations (deletions, point mutations, and translocations) were thought to be the basis for tumor formation *via* the inactivation of tumor suppressors and activation of oncogenes. Based on recent advances, however, epigenetic dysregulation, including aberrant patterns and levels of DNA methylation (DNAme) and post-translational modifications (PTMs) of histones, has emerged as another hallmark of cancer. For example, cancer cells usually exhibit global loss and regional gain of DNAme, which contribute to genomic instability and tumor suppressor silencing, respectively^2^. Mutations of histone-modifying enzymes and histone genes are also frequently observed in cancer, resulting in changes in histone modification patterns. The aberrant patterns can lead to inappropriate activation of oncogenes and/or inactivation of tumor suppressors, which synergistically contribute to tumor development and metastasis^3^. Thus, epigenetic dysregulation has an essential role in oncogenesis and tumor progression^4^.

DNAme is deposited on the fifth position of the cytosine base (5 mC) in mammalian cells, predominantly in the context of a CpG dinucleotide. Overall, 60%–80% of CpGs in the mammalian genome are methylated, mainly at repetitive sequences (satellite repeats, transposable elements, and sub-telomeres), gene bodies, and intergenic regions^5^. DNAme patterns are established by the *de novo* methyltransferases (DNMT3A and DNMT3B), aided by the catalytically-inactive accessory factor, DNMT3L, and maintained during DNA replication by the maintenance methyltransferase, DNMT1, and its regulator, UHRF1^5^. Conversely, removal of DNAme can be achieved by active demethylation *via* ten–eleven translocation (TET) dioxygenase-mediated oxidation of 5 mC or by passive dilution during DNA replication due to insufficient or deficient maintenance methylation^6^. Dozens of histone PTMs have been identified, including methylation, acetylation, phosphorylation, sumoylation, ubiquitylation, and ADP-ribosylation. Depending on the residues, valency and modification types, these histone PTMs inhibit or promote transcription. For instance, trimethylation of histone H3 on lysine 27 (H3K27me3) and H3K9me3 are associated with transcriptionally-inactive facultative and constitutive heterochromatin, respectively^7^. In contrast, promoters and enhancers are enriched with H3K4me3 and H3K27ac, respectively, for active transcription^8^. Additionally, euchromatic intergenic regions and actively-transcribed gene bodies are enriched with H3K36me2 and H3K36me3, respectively^9,10^.

Different epigenetic marks function in concert to regulate cellular processes. Some epigenetic marks have strong correlations, suggesting intrinsic relationships between them. In general, H3K4 methylation and DNAme are mutually exclusive, in part because H3K4 methylation disrupts the interaction between the ATRX-DNMT3-DNMT3L (ADD) domains of the DNMT3 family of proteins and the N-terminal tail of histone H3^11^. H3K27 methylation and DNAme are also usually anti-correlated, with DNAme appearing to antagonize the recruitment of polycomb repressive complex 2 (PRC2), which is responsible for H3K27 methylation^7^. In contrast, H3K9 methylation and DNAme are strongly associated, working synergistically to form heterochromatin for silencing genes and retrotransposons^7^.

Results from genome-wide studies suggest that H3K36me2/me3 have instructive roles in guiding *de novo* DNAme to intergenic regions and gene bodies, respectively^10,12^ (**Figure 1**). H3K36me2 is catalyzed by the NSD family of lysine methyltransferases (KMTs), which includes NSD1, NSD2, NSD3, and ASH1L, whereas H3K36me3 is deposited exclusively by SETD2. Mutations of these KMTs and DNMT3A or DNMT3B are often observed in human diseases, including cancer, and have overlapping features^7^. Of note, changes in DNAme patterns are a shared characteristic that may contribute to the etiology of these diseases. Sotos syndrome, caused by *NSD1* haploinsufficiency, and Tatton-Brown-Rahman syndrome, caused by *DNMT3A* germline mutations, are both childhood overgrowth disorders characterized by widespread loss of DNAme at intergenic DNA, macrocephaly, intellectual disability, and distinctive facial characteristics^13,14^. In this perspective, we discuss the interplay between H3K36me2/me3 and DNAme, the dysregulation of H3K36me2/me3 and DNAme in cancer, and potential therapeutic intervention strategies.

**Figure 1:**
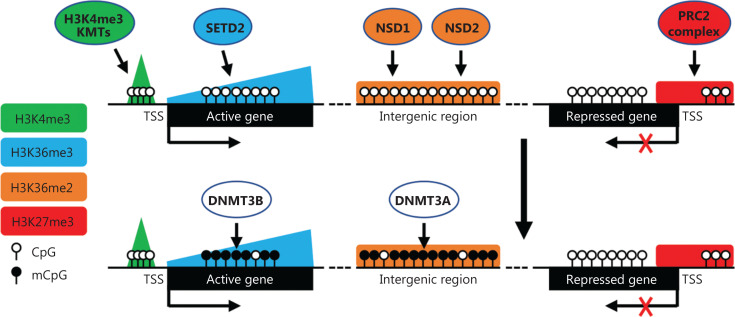
Summary of the instructive role of H3K36 methylation marks in guiding DNA methylation patterns. The major histone PTMs (color coded) that mark different genomic regions and the major modifying enzymes or complexes that “write” the PTMs are shown. The PWWP domains of DNMT3A and DNMT3B bind both H3K36me2 and H3K36me3, but with different affinities. As a result, *de novo* methylation of CpG sites in intergenic regions, enriched with H3K36me2, and gene bodies of actively-transcribed genes, enriched with H3K36me3, is preferentially catalyzed by DNMT3A and DNMT3B, respectively. TSS, transcription start site.

## H3K36me2/me3 recruit DNMT3A and DNMT3B

Biochemical, molecular, and structural analyses indicate that the proline-tryptophan-tryptophan-proline (PWWP) domain of DNMT3A shows dual recognition of H3K36me2 and H3K36me3, with a preferential affinity toward H3K36me2, directing DNMT3A to H3K36me2-enriched intergenic regions for *de novo* methylation^10,15,16^, whereas the PWWP domain of DNMT3B preferentially binds to H3K36me3, which is enriched in actively-transcribed gene bodies^12^. Genetic knockout of *Nsd1* in mouse embryonic stem cells (mESCs) or both *Nsd1* and *Nsd2* in mouse mesenchymal stem cells (MSCs) abrogates the recruitment of DNMT3A to H3K36me2-enriched intergenic regions and leads to a redistribution of DNMT3A to H3K36me3-modified gene bodies^10^. Reintroduction of wild-type NSD1, but not a catalytically-inactive form (C2023A), into *Nsd1*-depleted mESCs, results in restoration of global and intergenic levels of H3K36me2, as well as DNMT3A enrichment and DNAme at intergenic regions^10^. Most recently, NSD1 was shown to enrich active enhancers and act as a transcriptional coactivator independent of its catalytic activity in mESCs^17^. In contrast, depletion of SETD2-mediated H3K36me3 disrupts DNMT3B recruitment and *de novo* DNAme at H3K36me3-enriched gene bodies of actively-transcribed genes^12^. In addition, substitutions in the PWWP domain that impair the interaction between DNMT3B and H3K36me3 result in loss of DNAme in gene bodies^12^. DNMT3B-dependent intragenic DNAme has been shown to prevent gene bodies from spurious transcription initiation to ensure the fidelity of mRNA transcription initiation^18^. These results highlight the importance of the PWWP domains of DNMT3A/3B and the H3K36m2/me3 marks in orchestrating *de novo* DNAme in intergenic regions and gene bodies, respectively (**Figure 1**).

The role of H3K36 methylation in guiding *de novo* DNAme is also pronounced in the germline, with H3K36me2 and H3K36me3 showing sexually dimorphic functions. Depletion of SETD2 in oocytes leads to genome-wide loss of H3K36me3 and failure in establishing correct DNAme at gene bodies, which contributes to aberrant transcriptomes and defects in oocyte maturation and embryonic developmental arrest at the 1-cell stage^19^. SETD2 is dispensable for *de novo* DNAme in the male germline. Instead, NSD1-mediated H3K36me2 is critical for guiding DNMT3A-mediated *de novo* methylation in prospermatogonia, including at imprinted loci^20^. Notably, a subset of genes are silenced in *NSD1*-deficient sperm and *SETD2*-deficient oocytes due to invasion of H3K27me3 into territories normally occupied by H3K36me2/me3^19,20^. It is well-documented that H3K36 methylation antagonizes H3K27me3. H3K36me2/me3-modified nucleosomes have been shown to directly inhibit H3K27 methylation by PRC2 *in vitro*^21,22^. Thus, H3K36me2/me3-dependent *de novo* methylation functions in promoting normal transcription in germ cells of both genders.

## Dysregulation of the H3K36me2/me3-DNAme axis in cancer

The intimate relationship between H3K36me2/me3 and DNAme is apparent in cancer as well, as evidenced by the observation that tumors caused by mutations of H3K36 KMTs or the H3K36 residue are associated with aberrant DNAme. Recurrent H3K36 to methionine (H3K36M) or isoleucine (H3K36I) oncohistone mutations have been identified in a wide spectrum of human cancers, including 95% of chondroblastomas, undifferentiated sarcomas, and head and neck squamous cell carcinomas (HNSCCs)^23,24^, emerging as drivers of oncogenesis (**Table 1**). Although the H3K36M/I mutations are heterozygous and only occur in 1 of 16 genes coding for H3.1/2 or the variant histone (H3.3), H3K36M/I mutations dominantly affect the binding of several H3K36-specific KMTs, including NSD1/2 and SETD2, and inhibit their catalytic activities, resulting in decreases in the global abundance of the H3K36me2/me3 marks, loss of DNAme at intergenic regions and gene bodies, and gain of H3K27me3, especially at intergenic regions^9,38^. The H3K36M oncohistone mutation has been shown to block the differentiation of mesenchymal progenitor cells and generate undifferentiated sarcomas *in vivo*, mainly through the redistribution of H3K27me3 from PRC1 target genes to regions normally enriched with H3K36me2/me3^9^. A subsequent study has demonstrated that reduction in H3K36me2, but not H3K36me3, represents the common mechanism underlying tumorigenicity in cancers associated with dysregulation of H3K36 methylation. Specifically, genetic ablation of *NSD1* and *NSD2*, but not *SETD2*, is sufficient to recapitulate the H3K36M effects in several aspects, including enhancer activation, gene expression, differentiation blockade, and drug sensitivity^25^. Additionally, expression of H3K36M impairs adipogenesis in preadipocytes and cell proliferation in fibrosarcoma cancer cells, respectively, which can both be recapitulated by depleting NSD2, but not SETD2^39,40^. Furthermore, H3K36M/I mutations are not identified in cancer types with recurrent SETD2 mutations, such as clear cell renal cell carcinoma (ccRCC)^41^. In the absence of the antagonism by H3K36me2 and DNAme, the abnormal H3K27me3 distribution inhibits a subset of active enhancers localized in H3K36me2 domains. Together, these studies offer strong human genetic evidence supporting the conclusion that a decrease in H3K36me2 represents the key event in the development of tumors driven by the H3K36M oncohistone mutation. The H3.3 glycine 34-to-arginine/valine (H3.3G34R/V) substitutions were previously identified as somatic drivers in lethal cortical brain tumors of neuronal origin and pediatric glioblastomas^23,26–28^, while the H3.3G34-to-tryptophan (H3.3G34W) substitution was identified exclusively in bone tumors of mesenchymal origin, i.e., giant cell tumors of bone (GCTBs)^23^. The H3.3G34R mutation severely decreases H3K36me2 and impairs the recruitment of DNMT3A^42^, highlighting the dysregulation of the H3K36me2-DNAme axis in malignancy. Similarly, the H3.3G34W mutation found in GCTBs enhances tumor growth *in vivo* through promotion of aberrant PRC2 activity by blocking SETD2-mediated H3K36me3 at active enhancers^29^ (**Table 1**).

**Table 1:** Distinct human cancers caused by oncohistone variants, H3K36 methyltransferase and DNMT3A mutations

Gene/H3 residue	Mutations	Cancer types	Altered epigenome	References
*H3K36*	H3K36 to methionine (H3K36M) or isoleucine (H3K36I) mutations	Chondroblastomas, undifferentiated sarcomas, and head and neck squamous cell carcinomas (HNSCCs)	Global loss of H3K36me2/3, loss of DNAme at intergenic regions and gene bodies, and gain of H3K27me3, especially at intergenic regions	^23–25^
*H3.3G34*	H3.3 glycine 34-to-arginine/valine (H3.3G34R/V) mutations	Cortical brain tumors of neuronal origin, pediatric glioblastomas, and Tatton-Brown-Rahman syndrome	Reduction of H3K36me2 and DNAme at intergenic regions	^23,26–28^
	H3.3G34-to-tryptophan (H3.3G34W) mutation	Giant cell tumors of bone (GCTBs)	Decrease of H3K36me3 and increase of H3K27me3 at active enhancers	^23,29^
*NSD1*	Deletions and loss-of-function mutations	HNSCCs, and lung and cervical SCCs	Loss of H3K36me2 and DNAme at intergenic regions	^24,30,31^
*NSD2*	Elevated expression or hyperactive mutations (E1099K) as an oncogene	Multiple human cancers, including colorectal, prostate, and lung cancers	Increased level of H3K36me2	^32–34^
*SETD2*	Loss-of-function mutations	Multiple human cancers, including clear cell renal cell carcinoma (ccRCC), high-grade gliomas, and hematopoietic malignancies	Loss of H3K36me3	^35,36^
*DNMT3A*	Somatic heterozygous mutations (multiple), with R882 as a hotspot	Hematologic cancers, including acute myeloid leukemia (AML)	Loss of DNAme at many CpG sites throughout the genome	^7,37^

In addition to the oncohistone mutations that disrupt the interplay between H3K36me2/me3 and DNAme, recurrent mutations of H3K36 KMTs and DNMT3A are also identified in various types of cancer. For example, recurrent deletions and loss-of-function mutations affecting NSD1 are identified in 10%–15% of HNSCCs and lung and cervical SCCs^24,30^. *NSD1* mutations, which are mutually exclusive with the H3K36M mutation in HNSCCs, result in global DNA hypomethylation as a consequence of defective DNMT3A recruitment by H3K36me2 (**Table 1**). Together, the H3K36M and *NSD1* mutations define a molecularly- and clinically-specific HNSCC subgroup characterized by global depletion of H3K36me2, loss of DNAme, and gain of H3K27me3 at intergenic regions^24,31^. Somatic heterozygous mutations of *DNMT3A*, including a hotspot mutation affecting arginine 882 (R882) in the catalytic domain, are observed in acute myeloid leukemia (AML) and other hematologic malignancies. The R882 hotspot mutation has been shown to exert a dominant-negative effect to induce loss of DNAme at many CpG sites throughout the genome^37^ (**Table 1**).

While both NSD1 and NSD2 deposit H3K36me2, the roles of NSD1 and NSD2 in cancer appear to be distinct. Contrary to loss-of-function *NSD1* mutations in cancers, which suggest a tumor suppressor function, *NSD2* has emerged as an oncogenic gene with elevated expression or hyperactive mutations in multiple human cancers, including colorectal, prostate, and lung cancers. Ectopic expression of NSD2 rapidly accelerates malignant tumor progression, whereas silencing of NSD2 strongly attenuates tumor progression and metastasis in mouse models, mouse allografts, and human cancer cell lines^32–34^ (**Table 1**). The hyperactive variant, NSD2_E1099K_, cooperates with oncogenic KRAS signaling to drive lung adenocarcinoma (LUAD) pathogenesis, whereas CRISPRi-mediated NSD2 inactivation strongly attenuates tumor progression of LUAD^32^, indicating that the NSD2-H3K36me2 axis sustains oncogenic signaling and could be a *bona fide* LUAD therapeutic target.

Distinct *SETD2* mutations have also been identified across a wide range of human tumors, including ccRCCs, high-grade gliomas, and hematopoietic malignancies^35^, indicating tumor suppressor functionality. A recent study demonstrated that *SETD2* loss in ccRCCs promotes cancer metastasis, while H3K36me3 restoration greatly reduces distant metastases^36^ (**Table 1**).

## Epigenetic therapy targeting DNAme and H3K36 methylation for cancer treatment

Unlike genetic alterations, epigenetic changes are mostly reversible, making epigenetic therapy a promising approach for cancer treatment. The DNA demethylating agents, 5-azacytidine [5-azaC (Vidaza^®^)] and 5-aza-2′-deoxycytidine [5-aza-dC, decitabine (Dacogen^®^)], arguably the first FDA-approved epigenetic therapeutics, have been used for treating myelodysplastic syndrome (MDS), AML, and chronic myelomonocytic leukemia (CMML) (**Table 2**). The anti-tumor effects are partly due to the induction of “viral mimicry”^43,44^. Viral mimicry is characterized by the formation of cytoplasmic double-stranded RNAs (dsRNAs) derived from de-repression of endogenous retrovirus (ERV) transcripts, which provoke an interferon (IFN) response, thus boosting anti-tumor immunity^43,44^. Combination therapies using DNA demethylating agents and other epigenetic inhibitors, such as inhibitors against the H3K9 KMT G9A^45^ and histone deacetylases (HDACs)^46^, have been shown to enhance the viral mimicry effects (**Table 2**); however, 5-azaC and 5-aza-dC are cytidine analogs that incorporate into genomic DNA, causing substantial DNA damage and cytotoxicity, and are ineffective in treating solid tumors. Recently, GlaxoSmithKline (GSK) developed a new class of non-nucleoside, reversible DNMT1-selective inhibitors, which are superior to 5-aza-dC for tumor regression in a mouse model of AML^47^. In addition to inhibiting the catalytic activity of DNMT1, this class of compounds target DNMT1 for rapid degradation in cells^50^. Furthermore, non-nucleoside, reversible DNMT1-selective inhibitors are more potent in inducing hypomethylation and less toxic compared to cytidine analogs, making the non-nucleoside, reversible DNMT1-selective inhibitors potential therapeutic contenders (**Table 2**).

**Table 2:** Epigenetic inhibitors for cancer therapy

Inhibitor	Epigenetic target	Anti-tumor effects	Cancer treated	References
5-azaC and 5-aza-dC	DNA cytosine methyltransferases, including DNMT3A/3B and DNMT1	Induction of viral mimicry and tumor suppression	Hematological malignancies, including acute myeloid leukemia (AML), myelodysplastic syndrome (MDS), and chronic myelomonocytic leukemia (CMML)	^43,44^
GSK368502	DNMT1 specifically	Robust loss of DNA methylation and superior tumor regression with improved *in vivo* tolerability	AML	^47^
UNC0638	H3K9-KMT G9A	Combined with 5-aza-dC to further increase viral mimicry	Ovarian cancer cells	^45^
Trichostatin A (TSA)	Histone deacetylases (HDACs)	Combined with 5-aza-dC to further increase viral mimicry	Ovarian cancer cells	^46^
Tazemetostate	H3K27-KMT EZH2	Reactivation of IFN response, increased immune infiltration and inhibition of tumor growth	NSD1 inactivated squamous cell carcinomas (SCCs)	^48^
MS023	Type I PRMTs	Induction of dsRNA for viral mimicry response	Triple-negative breast cancer (TNBC)	^49^

The interplay between H3K36me2/me3 and DNAme offers new opportunities for developing therapeutic interventions against malignancy. A recent study indicates that *NSD1*-deficient SCCs unexpectedly exhibit a reduced IFN response and diminished tumor immune infiltration, despite high ERV expression^48^, making *NSD1*-deficient SCCs insensitive to DNA demethylating agents. *NSD1* inactivation leads to redistribution of H3K27me3 to regions normally occupied by H3K36me2 and DNAme, resulting in silencing of IFN response genes^48^. Indeed, treatment with tazemetostat, an inhibitor against EZH2, the major H3K27 KMT, leads to reactivation of the IFN response, increased immune infiltration, and inhibition of tumor growth^48^ (**Table 2**). Developing rational strategies to activate the viral mimicry response could be a general therapeutic approach for malignancies with distinct genetic and epigenetic landscapes. Inhibition of type I protein arginine methyltransferases (PRMTs) in triple-negative breast cancer (TNBC) by a small molecule compound (MS023) results in mRNA splicing alternation, dsRNA formation, and activation of the viral mimicry response, which exerts antitumor growth effects^49^ (**Table 2**). Additionally, vitamin C increases viral mimicry induced by 5-aza-dC to improve clinical efficacy in patients with myelodysplastic syndrome and leukemia^51^.

## Conclusions

Studies in recent years have provided compelling evidence for the instructive role of H3K36 methylation in shaping DNAme patterns. While DNMT3A and DNMT3B recognize both H3K36me2 and H3K36me3 *via* the PWWP domains, DNMT3A and DNMT3B have distinct preferences, with DNMT3A being mainly responsible for DNAme at H3K36m2-enriched intergenic regions and DNMT3B mainly for DNAme at H3K36me3-enriched gene bodies (**Figure 1**). H3K36me3/DNAme at gene bodies have important roles in alternative splicing, fidelity of mRNA transcripts, and genomic stability. In contrast, H3K36me2/DNAme at intergenic regions are usually associated with active enhancer activity by antagonizing H3K27me3 recruitment. Various human diseases, including cancer, have been linked to dysregulation of the H3K36 methylation-DNAme axis.

Despite the advances, research in this area is still in an early stage. Many fundamental questions remain to be explored. (1) What is the functional significance of chromatin marks in intergenic regions, gene bodies, and other genomic regions? (2) What are the determinants of the specificities of some histone-modifying enzymes, and the crosstalk among DNAme, H3K36 methylation, and other chromatin marks? (3) Why are interferon response genes preferentially silenced by the imbalance between H3K36me2 and H3K27me3 in NSD1-deficient SCCs? The underlying mechanisms remain elusive. Why do NSD1 and NSD2, both of which deposite H3K36me2 at intergenic regions, have the opposite roles in cancer? Specifically, NSD1 acts as an apparent tumor suppressor, whereas NSD2 primarily promotes oncogenesis. A better understanding of the interplay between H3K36 methylation and DNAme in normal and cancer cells would provide potential opportunities for novel therapeutic interventions. Conceptually, inhibiting lysine demethylases (KDMs) specific for H3K36me2, such as FBXL10 and FBXL11, is a potential strategy for treating cancers associated with loss-of-function *NSD1* mutations, and inhibiting NSD2 may be effective for NSD2-hyperactive cancers. Screening for NSD2 inhibitors or rationally developing cell-penetration capable proteolysis-targeting chimeras (PROTACs) for specific degradation of NSD2 protein will be promising drug design directions.

The anti-tumor effects of DNA demethylating agents and genetic ablation of *LSD1*, encoding a KDM specific for H3K4me1/me2, are related to induction of ERV expression, which triggers an IFN response^43,44,52^. A recent study suggested that the use of EZH2 inhibitors to overcome the low IFN response, despite DNA hypomethylation and de-repression of ERVs, in tumors associated with *NSD1* inactivation, thus further illustrating the benefits of elucidating the consequences when the H3K36 methylation-DNAme axis is disrupted or dysregulated^48^. Together, rationally developing strategies to boost viral mimicry response in different contexts could be a general therapeutic intervention to use against malignancy in our future endeavors.
